# Impact of tumour size measurement inter-operator variability on model-based drug effect evaluation

**DOI:** 10.1007/s00280-020-04049-5

**Published:** 2020-03-13

**Authors:** Aurélie Lombard, Hitesh Mistry, Sonya C. Chapman, Ivelina Gueoguieva, Leon Aarons, Kayode Ogungbenro

**Affiliations:** 1 Centre for Applied Pharmacokinetic Research, School of Health Sciences, Faculty of Biology, Medicine and Health, Manchester Academic Health Science Centre, University of Manchester, Stopford Building, Oxford Road, Manchester, M13 9PT UK; 2 Division of Pharmacy and Optometry, School of Health Sciences, Faculty of Biology, Medicine and Health, Manchester Academic Health Science Centre, University of Manchester, Manchester, M13 9PT UK; 3 Division of Cancer Sciences, School of Health Sciences, Faculty of Biology, Medicine and Health, Manchester Academic Health Science Centre, University of Manchester, Manchester, M13 9PT UK; 4 Eli Lilly and Company, Erl Wood Manor, Windlesham, UK

**Keywords:** Tumour size measurements, Inter-operator variability, Drug effect evaluation, Modelling

## Abstract

**Purpose:**

During oncology clinical trials, tumour size (TS) measurements are commonly used to monitor disease progression and to assess drug efficacy. We explored inter-operator variability within a subset of a phase III clinical trial conducted from August 1995 to February 1997 and its impact on drug effect evaluation using a tumour growth inhibition model.

**Methods:**

One hundred twenty lesions were measured twice at each time point; once at the hospital and once at the centralised centre. A visual analysis was performed to identify trends within the profiles over time. Linear regression and relative error ratios were used to explore the inter-operator variability of raw TS measurements and model-based estimates.

**Results:**

While correlation between patient-level estimates of drug effect was poor (*r*^2^ = 0.28), variability between the study-level estimates was much less affected (9%).

**Conclusions:**

The global evaluation of drug effect using modelling approaches might not be affected by inter-operator variability. However, the exploration of covariates for drug effect and the characterisation of an exposure–tumour shrinkage relationship seems limited by the high measurement variability that translates to a poor correlation of individual drug effect estimates. This might be addressed by the use of more precise computer-aided measurement methods.

**Electronic supplementary material:**

The online version of this article (10.1007/s00280-020-04049-5) contains supplementary material, which is available to authorized users.

## Introduction

Since 1979, several guidelines have been published to homogenise the report of response-to-treatment evaluation in oncology clinical trials, such as the World Health Organization (WHO) handbook [1] and the response evaluation criteria in solid tumours (RECIST) [2–4], giving tumour size (TS) measurements a central role in the assessment of drug efficacy. Indeed, even though overall survival remains the gold standard to characterise drug-induced patient benefit, several surrogate endpoints are based on TS measurements (e.g. progression-free survival, time-to-progression, objective response rate). Therefore, they contribute to decision-making during drug development and help accelerate experimental drug approvals by regulatory agencies [5, 6].

Tumour size measurements have been observed to be subject to inter-operator variability [7–9], which was identified by Thiesse et al*.* as one of the four reasons for misclassification of objective response rates, along with errors due to target lesion selection, technical imaging and coexistent diseases [10]. Because of its role during cancer drug development, any variability in tumour burden characterisation could lead to misinterpretation of clinical outputs and jeopardise the future and performance of patients within a trial, as well as the global evaluation of drug effect.

Mathematical modelling is increasingly used during drug development to analyse the sparse data collected during clinical trials and support go no–go decisions to the next phase. TS measurements have been used as an input in tumour growth inhibition (TGI) models to characterise drug effect for different cancer types, including non-small cell lung cancer [11]. Pharmacokinetic (PK) metrics have also been incorporated in TGI models as a covariate for tumour shrinkage to determine the exposure–response relationship, which could be used to perform adaptive dosing and tailor an individual effective dose for each patient based on tumour shrinkage [12]. However, there is limited knowledge about the impact of TS measurement inter-operator variability on model-based drug effect determination.

In this analysis, the inter-operator variability of TS measurements was explored in a subset of data of a multicentre phase III clinical trial. During this study, several computed tomography (CT) scans of individual lesions were measured by different operators at different sites: once at the hospital and once at the centralised centre. A TGI model was applied to these two sets of data separately to assess the correlation between parameter estimates and to address whether inter-operator variability affects the model-based drug effect evaluation at an individual and a population level.

## Materials and methods

### Tumour size measurement selection

The longest diameters of individual lesions were obtained from a phase III clinical trial where patients with stage IIIA, IIIB and IV non-small cell lung cancer received cisplatin alone or in combination with gemcitabine for up to six cycles [13].

Lesions were selected based on the availability of two measurements of the same CT-scan at each time point. One measurement was performed at the hospital where the patient was treated by different radiologists depending on the hospital location (local). The other one was measured at the centralised centre by a maximum of two radiologists (central). This analysis included a subset of 120 lesions of 62 (out of 522) patients representing 714 observations (357 paired measurements) with an average of 3 (1–5) time points per lesion and a median time of 59 (19–116) days between two measurements. At baseline, patients had a median of 2 (1–6) lesions and a median tumour size of 3.0 cm (0.25–13.0) and 2.8 cm (0.6–13.6) for the local and the central measurements, respectively. TS measurements were conducted according to the WHO criteria [1]; therefore, bidimensional measurements of tumour size were available. However, we decided to use the unidimensional longest diameter to mimic the RECIST criteria that are more commonly used since their publication in 2000 [2].

Almost half of the patients selected for this analysis had paired measurements only for one lesion out of the target lesions that were originally selected to evaluate response-to-treatment and therefore, the sum of the longest diameter (SLD) was not assessed in this analysis. Around 10% of the TS measurements were below the lower limit of quantification of 0.5 cm and were replaced by a value that is half the lower limit of quantification (0.25 cm).

### Correlation analysis

The local and the central profiles were visually compared to identify any trends and the percentage of lesion falling into specified patterns were derived. Profiles were classified as “similar” if the absolute difference between the local and the central measurements was equal or lower than the limit of quantification of 0.5 cm. If not, they were subjectively classified as “different but follow the same trend” if the two profiles seemed parallel over time and did not cross each other or as “different and did not follow the same trend” otherwise. The 62 patients selected for the analysis were spread across 28 study sites with a median of 1.5 patients per site (1–10); therefore, the study site effect on pattern classification could not be explored.

A tumour growth inhibition model was fitted to the local and the central data separately using Eq. 1 with an additive residual error component (NONMEM 7.3, first-order conditional estimation with interaction) to compute the population and the individual parameter estimates (Eq. 2). The models were assessed using goodness-of-fit plots and prediction-corrected visual predictive checks:

1 $${\text{TS}}\left( t \right) = {\text{TS}}\left( 0 \right) \times \left( {e^{{{\text{Kg}} \times t}} + { }e^{{ - {\text{Kd}} \times t}} - 1} \right),$$

where TS (*t*) is the tumour size profile over time, TS (0) is the tumour size at baseline, Kg is a growth rate constant, Kd is a decay rate constant.

2 $$P_{i} = \theta_{P} \times e^{{\eta_{i} }},$$

where $P_{i}$ represents the individual estimate of the parameter *P* for the lesion *i*, $\theta_{P}$ is the population estimate of the parameter *P*, $\eta_{i}$ is the random effect of the parameter *P* that is assumed to follow a normal distribution (0, $\omega^{2}$).

The correlation between the local and the central raw measurements at each time point and individual estimates of TS at baseline, growth rate constant and decay rate constant (drug effect) was explored by using linear regression analysis (R version 3.5.2 executed by the Rstudio interface version 1.1.453). Relative error ratios (RERs) were used to characterise the variation between the local and central raw data points, individual and population parameter estimates. Both the local and the central measurements contain variability. Therefore, on the basis that the “true” TS value should lie somewhere in between the two, we decided to apply a similar weight on the local and the central measurements, as suggested by Bland and Altman [14]. Thus, RERs were based on the mean local and central metrics (Eq. 3) and the limit of agreement (LOA) was derived using Eq. 4 [14]. This analysis did not assess whether the central measurement is more reliable for drug effect evaluation and disease progression characterisation, and whether it is more or less reliable for predicting OS.

3 $${\text{RER}} = \left( {{\text{local}} - {\text{central}}} \right) / {\text{mean}} \times 100,$$

where RER is the relative error ratio, local and central are the metrics derived from the TS measurements performed at the hospital and at the centralised centre, respectively, mean is the average local and central metrics:

4 $${\text{LOA}} = {\text{mean}} \; \pm \;2 \times {\text{SD}},$$

where LOA is the limit of agreement and SD is the standard deviation of the local and central metrics.

## Results

### Raw data correlation analysis

**Fig. 1:**
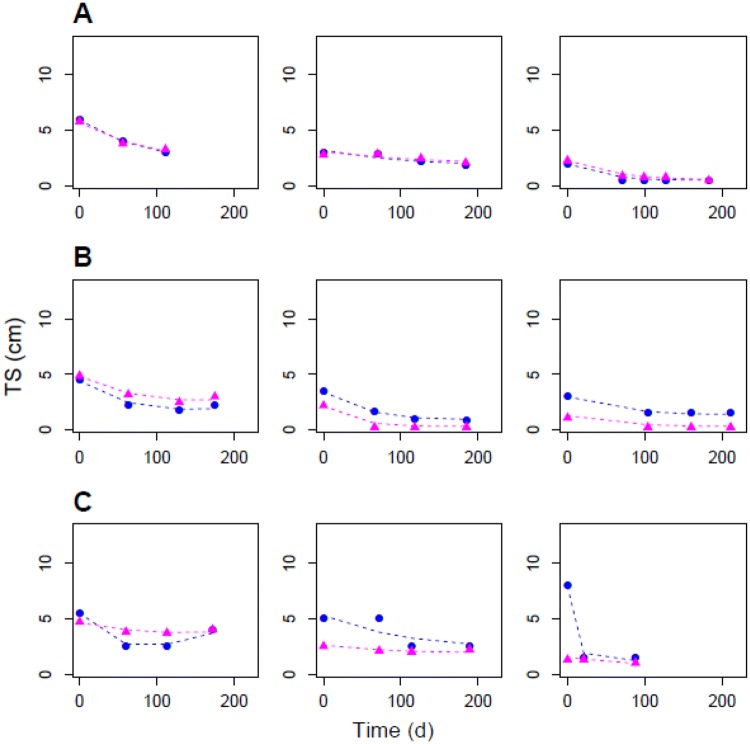
Individual fit plots of hospital (local) and centralised (central) measurements for selected individual lesions classified by patterns: **a** similar; **b** different but follow the same trend; **c** different and do not follow the same trend. The blue circles and the magenta triangles are the observations and the dashed lines are the individual model predictions for the local and the central measurements respectively. *TS* tumour size (individual longest diameter)

Three different patterns were visually observed within the local and the central profiles (Fig. 1); trends of the local and the central data (percent of data) were (1) similar over time (27.5%); (2) different but followed the same trend (40.0%); (3) different and did not follow the same trend (32.5%).

**Fig. 2:**
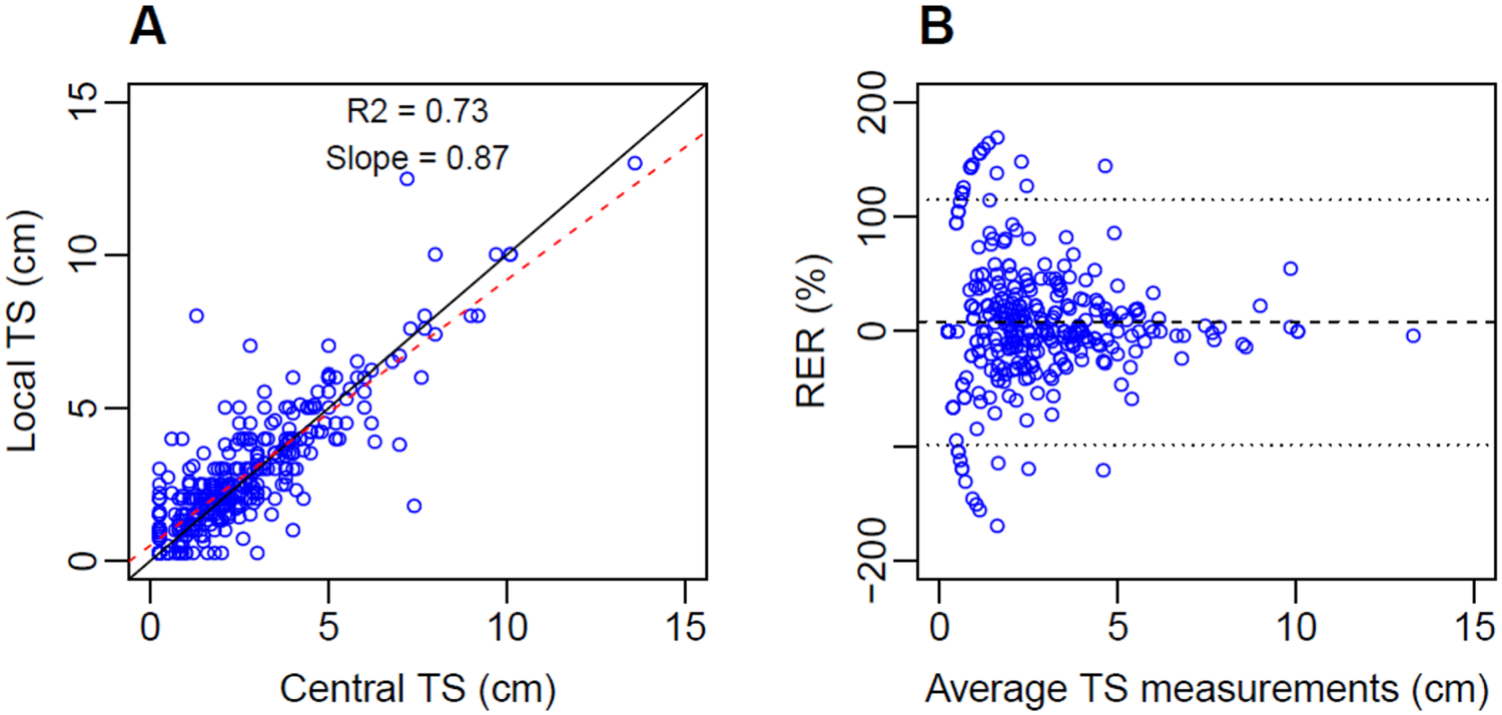
Correlation plot (**a**) and Bland–Altman plot (**b**) for hospital (local) and centralised (central) tumour size measurements at each time point. The open blue circles represent the observations. **a** The black line is the line of unity. The dashed red line is the linear regression line. **b** The black dashed line represents the mean of RERs. The two dotted black lines are the higher and the lower limit of agreement of RERs. *TS* tumour size (individual longest diameter), *RER* relative error ratio

The linear regression analysis revealed that the local and the central measurements at each time point were correlated with an *r*^2^ = 0.73 and the slope coefficient was equal to 0.87, relatively close to 1 (Fig. 2a). In 36 times out of 357 paired CT-scan measurements (10.1%), a lesion was considered non-existent (or below the quantification limit) by one operator while it was considered measurable by the other operator (0.5–3 cm). This occured at the last or/and before the last measurement, except for one baseline data point. Considering that a lesion non-measurable occurred 22 times (6.2%) for the centralised centre radiologist compared to 14 times (3.9%) for the hospital radiologist, the RERs were widely distributed from − 169.2 to169.2% (Fig. 2b). The LOA ranged from − 98.5 to 114.7%. The range between the 1st and the 3rd quantiles was narrower and distributed from − 14.0 to 25.0% around a median of 0%.

### TGI model output correlation analysis

The exponential growth and decay TGI model used to fit the TS measurements adequately described the local and the central profiles as shown on the representative individual fit plots (Fig. 1), on the goodness-of-fit plots and on the prediction-corrected visual predictive checks (see Online Resource).

The TGI model parameter estimates are presented in Table 1. The population parameters estimated using the local and the central set of observations were generally similar with RERs mostly below 10%. The local and the central tumour size at baseline estimates were particularly close (RER = 6%). The local drug effect (Kd) was slightly higher than the central population estimate (RER = 9%). The growth rate constant (Kg) was the only parameter for which the local and the central estimates differed by more than 10% (RER = 28%).

**Table 1:** Tumour growth inhibition (TGI) model parameter estimates for the local and the central sets of observations

Parameters (units)	Estimates (% RSE)		RERs
	Local set	Central set	
TS(0) (cm)	3.1 (5%)	2.9 (6%)	6%
Kg (/d)	0.0012 (15%)	0.00091 (27%)	28%
Kd (/d)	0.0077 (11%)	0.0070 (15%)	9%
IIV—TS(0)	0.31 (14%)	0.33 (12%)	–
IIV—Kg	0.20 (33%)	0.43 (28%)	–
IIV—Kd	0.44 (31%)	0.59 (28%)	–
Additive residual error (cm)	0.43 (9%)	0.40 (12%)	7%

*TS(0)* tumour size at baseline, *Kg* growth rate constant, *Kd* decay rate constant, *IIV* inter-individual variability, *RSE* relative standard error, *Local set or Central set* hospital or centralised centre observations used for parameter estimation, respectively, *RERs* relative error ratios of the local–central difference compared to the mean between the local and the central population parameters

**Fig. 3:**
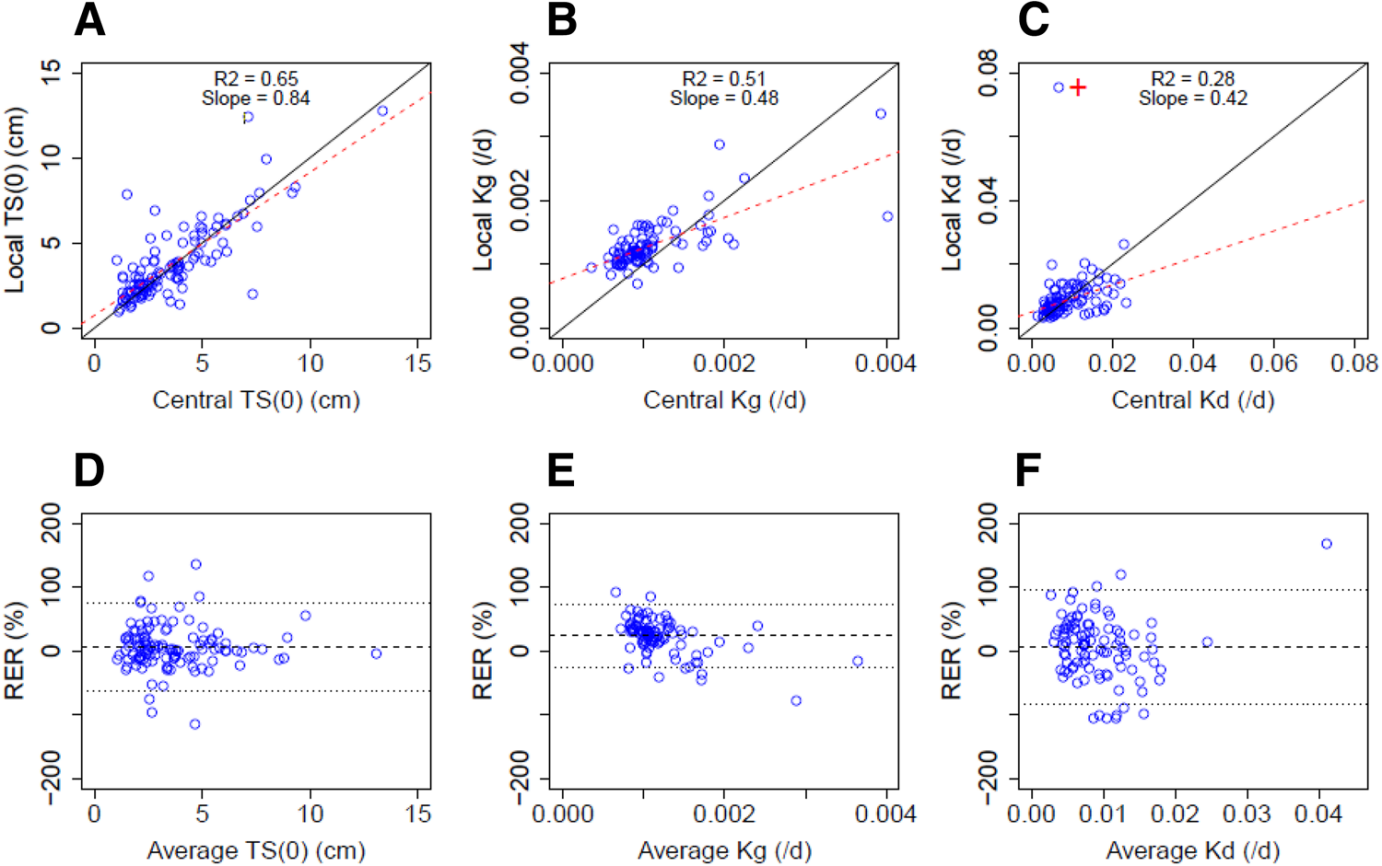
Correlation plots (**a**–**c**) and Bland–Altman plots (**d**–**f**) for hospital (local) and centralised (central) individual tumour size at baseline (**a**, **d**), growth constant rate (**b**, **e**) and drug effect estimates (**c**, **f**). The open blue circles represent the individual estimates. **a**–**c** The dashed red line is the linear regression line. The black line is the line of unity. **d**–**f** The black dashed line represents the median of RER. The two dotted black lines are the higher and the lower limit of agreement of RERs. **c** The open circle marked with a red cross was considered as an outlier and was not included for the linear regression analysis. *TS(0)* the tumour size at baseline, *Kg* growth rate constant, *Kd* decay rate constant, *RER* relative error ratio

The linear regression analysis showed that the local and the central individual tumour size at baseline parameter estimates were correlated with an *r*^2^ = 0.65 and with a slope coefficient of 0.84, relatively close to 1, similar to the raw measurement (Fig. 3a). The correlation between the local and the central individual estimates of Kg was also good (*r*^2^ = 0.51); however the slope coefficient was low (slope = 0.48). Mostly, the local Kg individual estimates were higher than the central Kg estimates. Very few data were on the line of unity and the 1st and 3rd RER quartiles did not include 0 (15.3%, 34.1%, median = 27.7%) (Fig. 3b). Surprisingly, the local and the central individual estimates of drug effect were poorly correlated (*r*^2^ = 0.28) (Fig. 3c). As for Kg, the slope coefficient of Kd estimates was low (slope = 0.42); however, individual estimates were closer to the line of unity and the 1st and 3rd RER quartiles did include 0 (− 18.0%, 31.3%, median = 9.3%). The LOA of the model parameter estimates were lower than the LOA of the raw measurements, being (− 63.1%, 73.7%), (− 25.3%, 72.6%) and (− 83.9%, 96.6%) for tumour size at baseline, Kg and Kd estimates, respectively.

## Discussion

Tumour burden has a central role in response-to-treatment evaluation of solid tumours during oncology clinical trials, as it is used to generate surrogate endpoints for overall survival such as progression-free survival or objective response rates, which are used to ascertain drug effect and gain regulatory approval for marketed medicines. Recently, TS measurements have been used as an input in tumour growth inhibition models that allow the determination of drug effect on the longitudinal TS kinetics and have been coupled with survival analysis to investigate early predictors of drug efficacy [11, 15–17]. Variability on TS measurements has been observed with different cancer types, including non-small cell lung cancer [8, 9, 18–20], and has led to misinterpretation of response-to-treatment [8–10]. In the current analyses, we explored the inter-operator variability of TS measurements in a selected population of a phase III clinical trial and its impact on model-based drug effect evaluation at the individual lesion level (individual longest diameter).

The visual data exploration has shown that different patterns were present within the hospital (local) and the centralised centre (central) measurement profiles (Fig. 1), which might affect the evaluation of drug efficacy in various ways. For 27.5% of the data, the local and the central measurements were similar at each time point and so no impact would be observed on drug effect assessment. For 40.0% of the measurements, the local and the central profiles were different, but did follow a similar trend over time and so the difference would mostly affect the estimation of tumour size at baseline from a modelling perspective. In addition, the fact that one lesion at baseline was considered non-existent by one radiologist and measurable by the other one could lead to different target lesion selection. As it has been previously observed [8–10], it could also affect the determination of surrogate endpoints for overall survival that are based on TS, such as the objective response rate and PFS. For the objective response of target lesions, the RECIST criteria defined partial response and disease progression based on the percentage of change in SLD (partial response: − 30% from baseline; disease progression: +20% and at least 5 mm from the lower SLD value) [2]. Therefore, even with a similar pattern, the percentage change in TS from baseline (or from the lower SLD value) would differ according to absolute SLD value and will be larger for the smaller measurements, potentially leading to different objective response classification. For 32.5% of the lesions, the local and the central measurements were different and did follow a different pattern and so the difference would directly affect the estimation of the drug effect using TGI models, as well as the objective response classification.

The linear regression analysis has shown that the local and the central measurements at each time point were correlated with an *r*^2^ = 0.73 (Fig. 2a). This could be considered as a good-to-strong correlation between two distinct variables [21]. However, in this case, where the two analysed variables are derived from the same CT-scan and therefore, are assumed to take the same “true value”, the only source of variability relies on the operator measurement (mostly representing measurement method and human interpretation) and thus a higher correlation between the two measurements would have been expected. The variation between the local and the central data seemed to decrease with the increase of the absolute tumour size, as previously reported [22]. The RERs were widely distributed from − 169.2 to 169.2% (Fig. 2b). Higher ratios were mostly observed for TS values smaller than 3 cm and when one radiologist measured a tumour that was considered non-existent or below the quantification limit of 0.5 cm by the other radiologist. The LOA ranged from − 98.5 to 114.7%, which is particularly high compared to other published results on unidimensional measurement inter-observer variability, such as the meta-analysis of Yoon et al. reporting a range of − 22.1 to 25.4% [22]. The current study was conducted from August 1995 to February 1997; therefore, the part of the inter-observer variability that is due to measurement tools would be assumed to have been reduced over the years due to technical progress, allowing a more precise measurement of small tumours. The larger variability in TS measurements might also be due to the fact that this study was not designed to assess inter-observer variability and therefore the radiologists may have had a different mindset when making the measurements. The distribution between the 1st and the 3rd quantiles of the RERs was narrower and was distributed mostly from − 14.0 to 25.0%, closer to the variation observed by Yoon et al. for ILD [22]. It has been suggested that TS measurements performed at the hospital where the patient was treated would tend to underestimate disease progression because of the benevolence of the doctor for his patient [9]. It was not the case in this study, as the RERs median was equal to zero, indicating that the discrepancies between the two measurements were not systematically biased.

The linear regression analysis of the modelling outputs has shown that the local and the central individual estimates of TS at baseline were correlated, similar to the raw measurements (Fig. 3a). There was a correlation between the local and the central individual estimates of Kg (*r*^2^ = 0.51); however, the slope coefficient was low (slope = 0.48). Mostly, the local Kg individual estimates were higher than the central Kg estimates, very few data points were on the line of unity and the 1st and 3rd RER quartiles did not include 0 (15.3%, 34.0%, median = 27.7%) (Fig. 3b). This suggests that the Kg parameter is particularly sensitive to inter-operator variability at an individual level and might not be reliable. Surprisingly, the local and the central individual estimates of drug effect were poorly correlated (*r*^2^ = 0.28) (Fig. 3c), indicating that the variability induced by the operators when measuring TS has a direct impact on the estimation of drug effect at an individual level when using such precise and data-dependent approaches as TGI models. The fact that different responses were observed for the same lesion, exposed to a certain drug level also suggests that the determination of the exposure–tumour shrinkage relationship might be affected by inter-operator variability. Therefore, to date, TS measurements might not be sufficiently precise for use in adaptive dosing approaches where individual effective dose are tailored for each patient based on tumour shrinkage [12]. In this analysis, we did not assess the level of precision that would be needed to perform adaptive dosing approaches. As for Kg, the slope coefficient of Kd estimates was low (slope = 0.42); however, individual estimates were closer to the line of unity and the 1st and 3rd RER quartiles did include 0 (− 18.0%, 31.3%, median = 9.3%). The TGI model allows the estimation of a residual error and therefore, the identification of the “true” tumour size profile, which results in lower LOAs for the parameter estimates, being (− 63.1%, 73.7%), (− 25.3%, 72.6%) and (− 83.9%, 96.6%) for tumour size at baseline, Kg and Kd estimates, respectively, compared to the LOA of the raw measurements (− 98.5 to 114.7%) (Fig. 3d–f). This suggests that using TGI model might be slightly more precise than raw data.

The population parameter estimates were found to be mostly similar with less than 10% variation between the local and the central estimates, including for the drug effect estimate (Kd) where only 9 days difference between the local and the central halving time (3.0 and 3.3 months, respectively) was observed. This suggests that drug-response interpretation for a typical patient would be close and that the evaluation of the drug effect at a population level might be similar. This is partially reassuring as these TGI models are mainly used to assess the global effect of a cancer drug rather than individual profiles during drug development. However, individual profiles are used to explore potential covariates (e.g. drug exposure) and give further insight on drug effect. Therefore, as long as TS measurements are not precise enough, the trust that we can have on the relationship between covariates and tumour shrinkage is compromised and thus, information provided by TGI models are limited for this application. TGI models are still informative of drug effect on target lesions; however, this limitation needs to be kept in mind, especially when TGI models are used with dosing optimisation purposes. Among the population parameter estimates, only the growth constant rate was very different depending on the observation sets, with 6 months difference between the tumour size doubling time (19 months and 25 months for the local and the central data, respectively). This suggests that the Kg parameter might also be particularly sensitive to inter-operator variability at a population level. There are different potential reasons why Kg seems more affected by inter-operator variability that Kd. It might come from the structure of the bi-exponential model used to describe TS profiles that results in a correlation between Kd and Kg making the parameters more difficult to identify. It might also be due to the fact that generally, the estimation of Kg relies only on two time points, as patients will be withdrawn from the treatment after progression and imaging will stop, whereas more time points will be available to characterise Kd in case of response, making Kd estimation more robust. The growth rate constant has been identified as a biomarker for drug efficacy [23, 24]; however, its sensitivity to inter-observer variability might suggest that it could not be a parameter of choice for OS prediction. Therefore, this parameter should be used carefully, especially as it has been suggested that Kg might not be linked to patient survival post-progression [25].

This analysis was conducted using ILD rather than SLD (RECIST criteria) as almost half of the patients selected for this analysis had paired measurements for one tumour only. The meta-analysis conducted by Yoon et al. has suggested that inter-operator variability might slightly be reduced when computing the SLD rather than ILD reporting a 95% LOA ranging from − 19.2 to 19.5% for SLD and from − 22.1 to 25.4% for ILD [22]. However, this decrease in LOA does not necessary lead to a better agreement on the objective response categorisation, as the variability due to the imaging methods would be added on top [20, 26]. Indeed, Erasmus et al. observed an increase in misclassification when using SLD compared to ILD (increasing from 30 to 31% for disease progression and from 3 to 15% for partial response) [8].

In 1984, Warr et al. shared their concerns about the variability in TS measurements being close to the objective response thresholds of the WHO criteria, especially for the determination of disease progression of target lesions [9]. These concerns have been partially taken into account in 2000 by the first release of the RECIST criteria, which uses the more conservative threshold of +20% of the SLD for disease progression. Assuming spheroid tumours, +20% of the SLD would correspond to +44% of the sum of the products of the longest diameters, which is higher than the +25% of the sum of the products of the longest diameters originally recommended by the WHO criteria [2]. However, this threshold of +20% might not be sufficiently restrictive, as the variability in TS measurements can exceed this value when (1) the tumour edge is not well defined, (2) when only one target lesion is selected, or/and (3) when different radiologists perform the measurements over time (inter-observer is typically higher than intra-observer variability) [8, 22]. Therefore, it would be interesting to consider new thresholds that would be more likely to account for this variability in TS measurements, notably for disease progression categorisation, as suggested by Warr et al. [9]. Otherwise, efforts should be made to generalise the use of computer-aided TS measurements, which have shown to considerably reduce variability in TS measurements [19] or artificial intelligence, which have shown to outperform radiologist in the screening of breast cancer [27]. Rather than categorisation, the use of a longitudinal approach like TGI models should be considered as it allows the analysis of the entire TS profile. Even though these approaches are particularly dependent on the data, they do allow the estimation of a residual error and the identification of a “true” value for tumour size and appear to give lower LOAs on parameters compared to the LOA on raw measurements. However, as long as TS measurements are not precise enough, the exploration of covariates for tumour shrinkage is limited, as is also the characterisation of the exposure–response relationship. This might be addressed by the use of more precise computer-aided measurement methods. TGI models can still be informative of drug effect; however, this limitation needs to be kept in mind, especially when TGI models are used with dosing optimisation purposes. There may be other biomarkers of drug efficacy which may be more appropriate, such as circulating cancer cells, circulating tumour DNA or neutrophil counts.

In summary, this analysis confirmed that the operator is an important aspect in the variability in tumour size measurements. This variability could affect the individual model-based interpretation of drug response as well as the characterisation of exposure–response relationship, as poor correlation was observed between the local and the central individual drug effect estimates. However, drug-response interpretation for a typical patient will be similar as population parameter estimates were comparable; suggesting that the global evaluation of drug efficacy by modelling approaches might not be affected.

## Electronic supplementary material

Below is the link to the electronic supplementary material.

Supplementary file1 (DOCX 528 kb)
