# Supplementary material for: Impact of tumour size measurement inter-operator variability on model-based drug effect evaluation
Source: Cancer Chemother Pharmacol. 2020 Mar 13;85(4):817–25. doi: 10.1007/s00280-020-04049-5 (PMC7125250; doi:10.1007/s00280-020-04049-5)

**Online Resource**

**Impact of tumour size measurement inter-operator variability on model-based drug effect evaluation**

Aurélie Lombard^1,2^, Hitesh Mistry^2,3^, Sonya C. Chapman^4^, Ivelina Gueoguieva^4^, Leon Aarons^1,2^, Kayode Ogungbenro^1,2^

^1^Centre for Applied Pharmacokinetic Research, ^2^Division of Pharmacy and Optometry, ^3^Division of Cancer Sciences,

School of Health Sciences, Faculty of Biology, Medicine and Health,

Manchester Academic Health Science Centre,

University of Manchester, Manchester M13 9PT, UK

^4^Eli Lilly and Company, Erl Wood Manor, Windlesham, United Kingdom

**Corresponding author** Aurélie Lombard

Tel: 01613062375

Email: aurelie.lombard@postgrad.manchester.ac.uk

Postal address: Stopford Building, University of Manchester, Oxford Road, M13 9PT, UK

Individual local (blue) and central (magenta) tumour size measurement profiles over time for all selected lesions. Tumour size is indicated in centimetres.


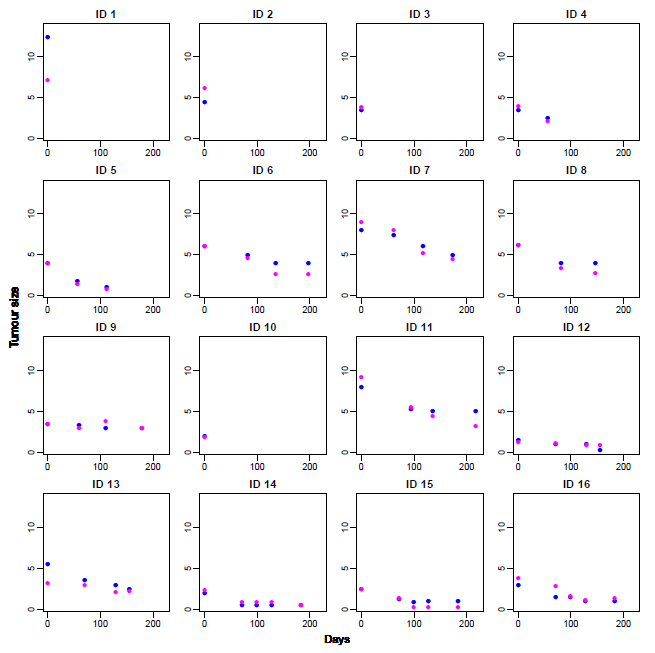


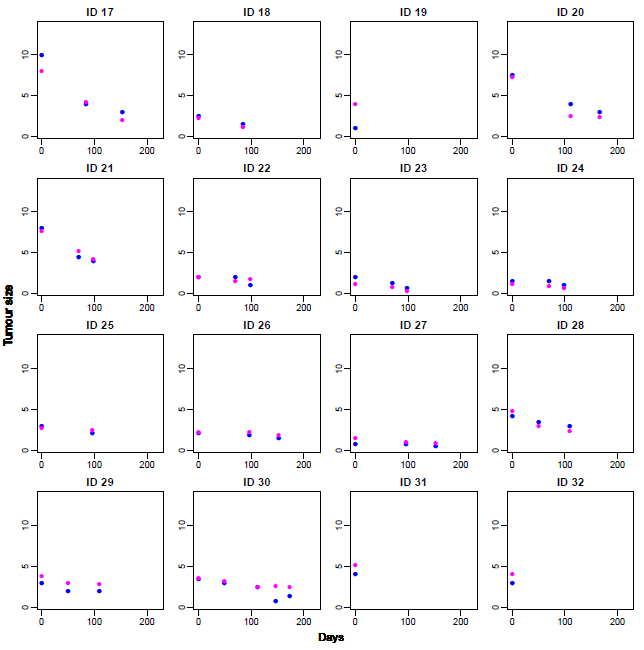


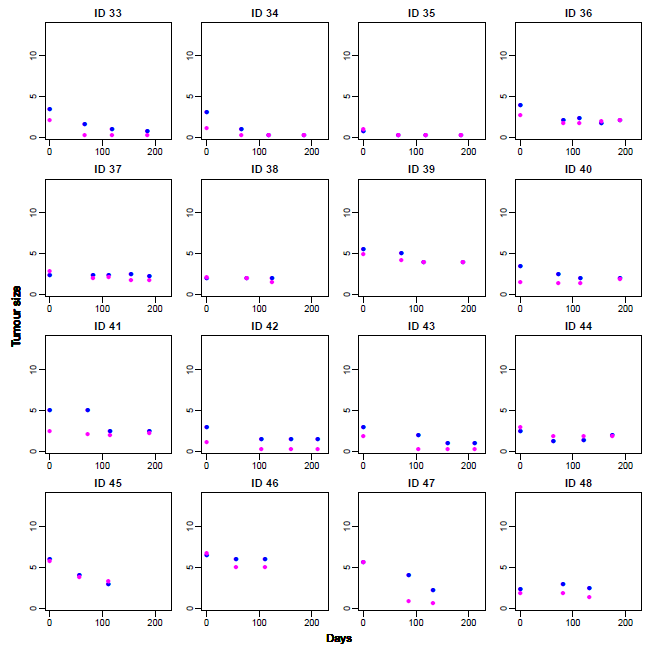


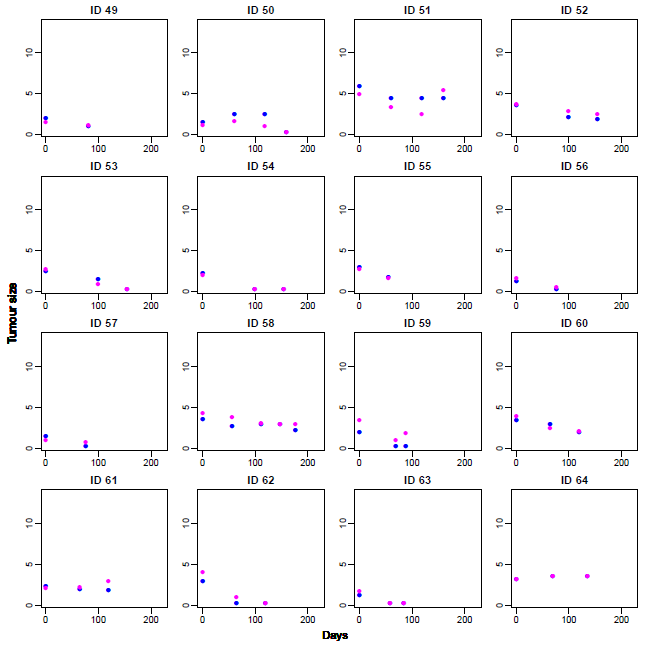


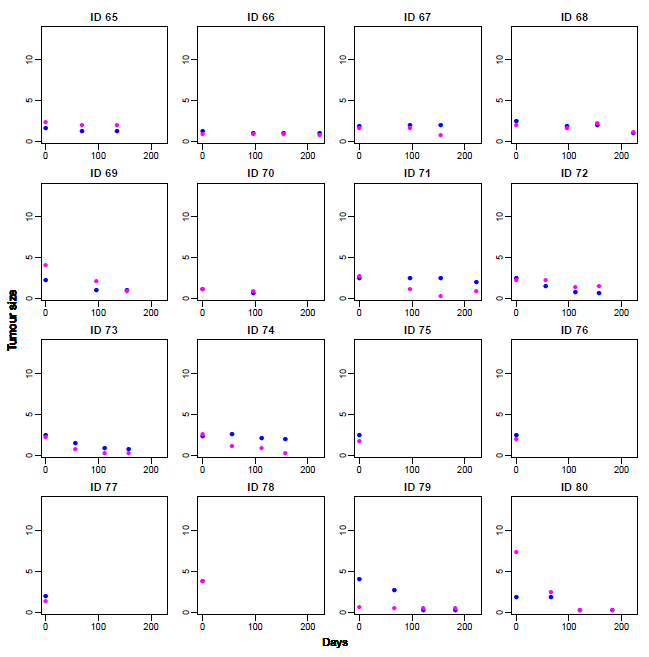


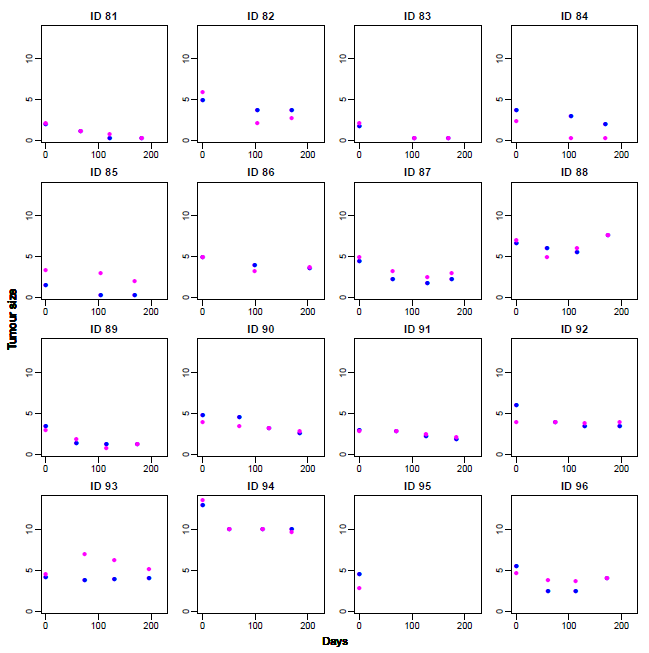


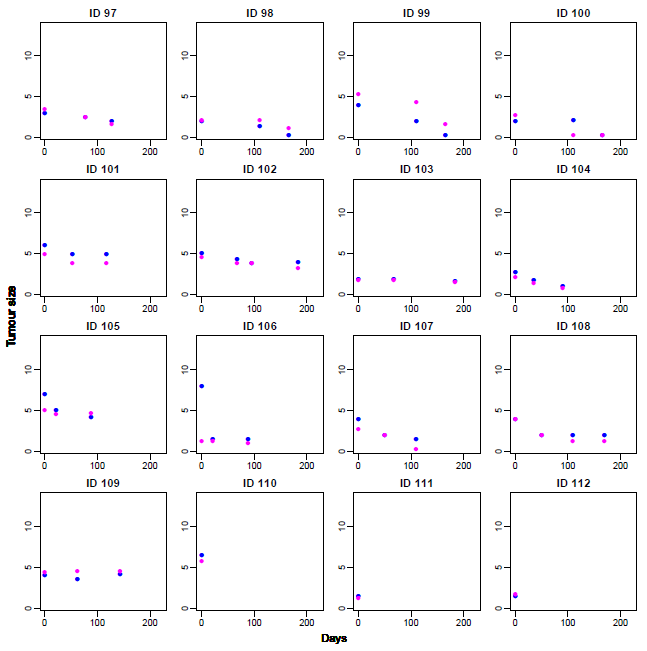


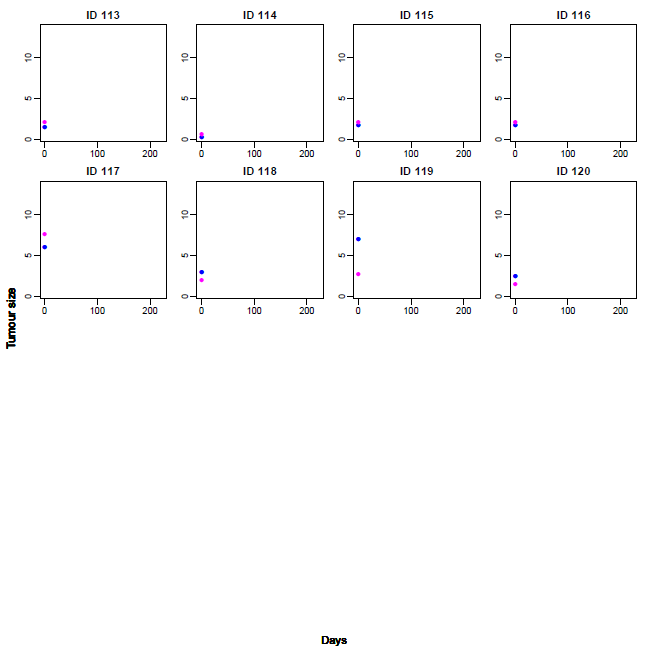


Goodness-of-fit plots of the TGI model using the hospital (Local) (**a**) or the centralised (Central) (**b**) set of observations. Time is in day units. IWRES are the individual weighted residuals. CWRES are the conditional weighted residuals. The black line is the line of unity. The dashed red line represents a linear regression line


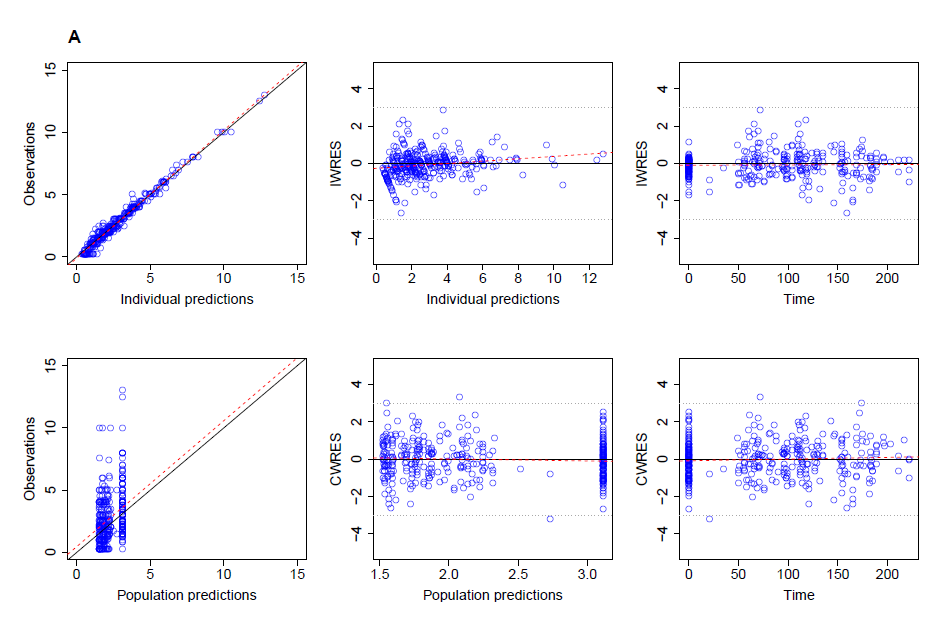


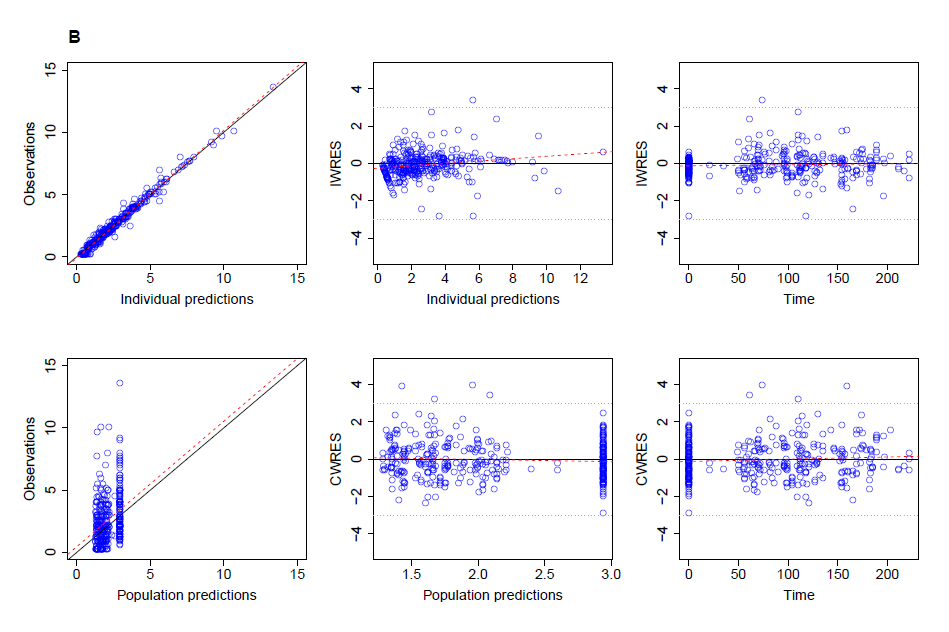


Visual predictive checks for the TGI model using the hospital (Local) (**a**) or centralised (Central) (**b**) set of observations. Time is in day units. (Upper section) The open blue circles are the observations (individual longest diameter). The plain and the two dashed red lines are the median, the 5^th^ and the 95^th^ percentiles of the observations, respectively. The red and blue areas are the 95% confidence interval of the prediction median, 5^th^ and 95^th^ percentiles, respectively. (Lower section) The line represents the proportion of observations that are below the lower quantification limit over time. The blue area is the 95% confidence interval of the predicted proportion of value below the limit of quantification

**A B**


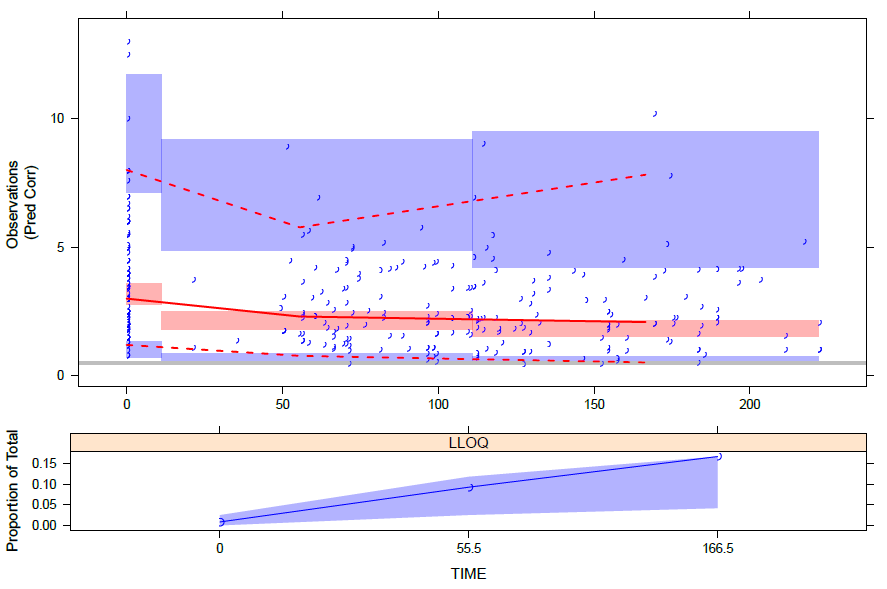

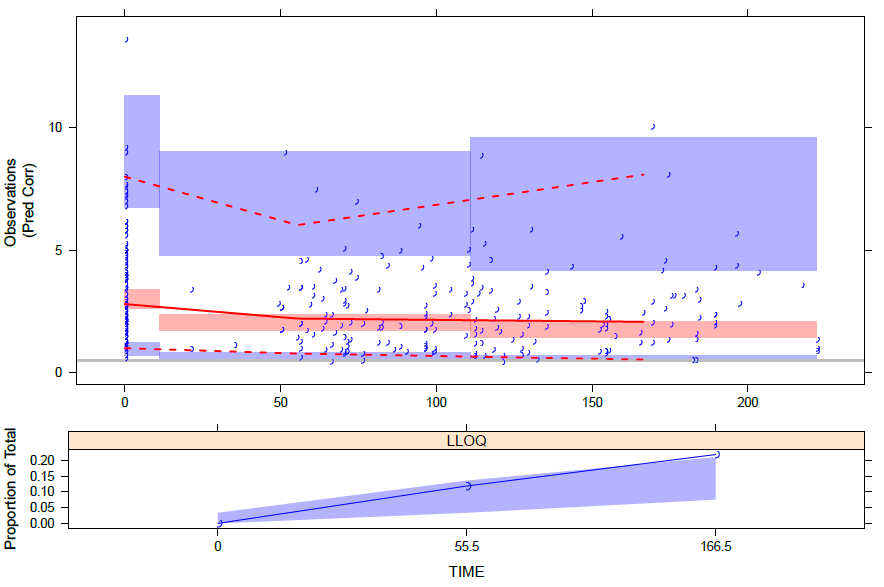

Supplement: Supplementary file 1 — Supplementary file1 (DOCX 528 kb) [file 280_2020_4049_MOESM1_ESM.docx]
